# The Type III Secretion System (T3SS) of *Chlamydophila psittaci* Is Involved in the Host Inflammatory Response by Activating the JNK/ERK Signaling Pathway

**DOI:** 10.1155/2015/652416

**Published:** 2015-01-22

**Authors:** Qing-zhi He, Huai-cai Zeng, Yan Huang, Yan-qun Hu, Yi-mou Wu

**Affiliations:** ^1^Pathogenic Biology Institute, University of South China, Hengyang 421001, China; ^2^Department of Parasitology, Zhongshan School of Medicine, Sun Yat-sen University, Guangzhou 510275, China

## Abstract

*Chlamydophila psittaci* (*C. psittaci*) is a human zoonotic pathogen, which could result in severe respiratory disease. In the present study, we investigated the role and mechanism of the type III secretion system (T3SS) of *C. psittaci* in regulating the inflammatory response in host cells. *C. psittaci*-infected THP-1 cells were incubated with the specific T3SS inhibitor INP0007, inhibitors of ERK, p38, or JNK, and the levels of inflammatory cytokines were analyzed using Q-PCR and ELISA. The levels of ERK, p38, and JNK phosphorylation were analyzed by Western blot. Our results verified that INP0007 inhibited chlamydial growth *in vitro*, but the coaddition of exogenous iron completely reversed the growth deficit. INP0007 inhibited the growth of *C. psittaci* and decreased the levels of IL-8, IL-6, TNF-*α*, and IL-1*β*. Exogenous iron restored the chlamydial growth but not the production of inflammatory cytokines. These results demonstrated that the expression of inflammatory cytokines during infection was associated with the T3SS which reduced by incubation with ERK and JNK inhibitors, but not with p38 inhibitor. We concluded that the T3SS elicited inflammatory responses by activating the JNK or ERK signaling pathways in the infection of *C. psittaci*.

## 1. Introduction

*Chlamydophila psittaci* (*C. psittaci*) is an intracellular pathogen of birds and a human zoonotic pathogen.* C. psittaci* primarily infects birds, and the transmission of* C. psittaci* from birds to human often results in severe respiratory disease [1–4]. Parrots are one of the most popular pet birds in China and can harbour* Chlamydia* which has significance for human and animal health. The occurrence of* C. psittaci* in China gives rise to potential environmental contamination with Chlamydiaceae and a public health concern [5].* C. psittaci* exhibits a biphasic developmental cycle and replicates in vacuoles in the cell cytoplasm of eukaryotes to form inclusion bodies [6]. In the host cell,* C. psittaci *secretes toxic proteins called the effector molecules of the type III secretion system (T3SS) to induce damage. The effector proteins of the T3SS appear to be temporally transcribed during the early (1.5–8 h after infection), middle (12–18 h after infection), and late (by 24 h) stages of the developmental cycle [7]. These stages are consistent with the development of* C. psittaci* from a nonreplicating infectious particle (elementary body, EB), its progression to an intracellular replicating body (reticulate body, RB) and finally, RB proliferation and RB to EB redifferentiation, respectively.

Chlamydiae modify their inclusion bodies to avoid lysosomal degradation and escape the host endocytic pathway. The T3SS has been hypothesized to play an important role in this process. The T3SS is a syringe-like structure that consists of large number of proteins and facilitates the targeted secretion of effector proteins directly into the host cytosol [8]. This system is highly conserved among various bacterial species. T3SSs have been shown to be involved in the inflammation induced by infections of* Salmonella *spp. [9],* Yersinia *spp. [10],* Shigella *spp. [11, 12],* Pseudomonas *spp. [13–15],* Burkholderia *spp. [16], and* Chlamydia trachomatis *[17], and T3SSs have also been shown to be necessary for cytokine secretion. Inflammatory cytokines such as interleukin (IL)-8 and IL-6 are upregulated in cells of the cervical epithelioid carcinoma HeLa cell line and the cervical squamous carcinoma SiHa cell line after infection with* C. psittaci* [18]. These studies highlighted the rationale for determining if the T3SS of* C. psittaci* was able to modulate the host inflammatory response.

INP0007 (a T3SS inhibitor) can inhibit the secretion of specific effector proteins of the T3SS of* Yersinia *spp. [19],* Salmonella *spp. [20], and* Chlamydia muridarum* [17] and can also inhibit the growth and development of* C. muridarum* [21] and* Chlamydia pneumoniae* [22]* in vitro*. The inhibitory effect of INP0007 can be attenuated by the addition of exogenous iron [17, 22].

The mitogen-activated protein kinase (MAPK) signaling pathway, which includes extracellular signal-regulated kinase (ERK), p38, and c-Jun N-terminal kinase (JNK), modulates the activities of proinflammatory transcription factors and induces the production of cytokines. Previous studies have demonstrated that MAPKs contribute to cytokine production during chlamydial infections [23, 24]. Based on the involvement of T3SS in the inflammatory responses of other pathogenic bacteria, in the present study, we investigated the role of the T3SS in cytokine production during* C. psittaci* infection as well as the pathway involved in this process.

## 2. Materials and Methods

### 2.1. Propagation of* C. psittaci*

*C. psittaci* serovar 6BC were propagated in HeLa 229 cell monolayers, as described previously [18]. Briefly, HeLa 229 cells were grown in RPMI-1640 medium (Sigma-Aldrich, Poole, UK) with 10% fetal bovine serum (FBS, Invitrogen, Paisley, UK) at 37°C in 95% air/5% CO_2_. Cultures infected with* C. psittaci* serovar 6BC were cultivated for 44 h before harvesting. Infected monolayers were detached with 0.25% trypsin/EDTA and then pelleted and sonicated to lyse the host cells. Cell debris was removed by differential centrifugation. Chlamydial EBs were pelleted, resuspended in SPG buffer (250 mM sucrose, 10 mM sodium phosphate, 5 mM L-glutamic acid), and frozen at −80°C. Infectious titers were determined by titration on HeLa 229 cell monolayers and staining with a FITC-labeled monoclonal antibody against chlamydial LPS (Meridian Diagnostics, Inc., Cincinnati, OH) and are expressed in inclusion-forming units (IFUs).

### 2.2. Culture and Treatment of Human Leukemia Cell Line (THP-1)

THP-1 cells were purchased from the Type Culture Collection of the Chinese Academy of Sciences (Shanghai, China) and cultured routinely in RPMI-1640 medium with 10% FBS, 50 IU/mL penicillin, and 50 *µ*g/mL streptomycin. PCR was used to exclude mycoplasma contamination in the cell lines and in the* C. psittaci* stock solution.

In our experiments, THP-1 cells were seeded onto 13 mm glass coverslips in 24-well plates at a density of 1.0 × 10^5^ cells per well in RPMI-1640 medium containing 10% FBS. The cells differentiated into macrophages following the addition of 100 ng/mL phorbol 12-myristate 13-acetate (PMA) for 24 h. The medium was discarded to remove nonadherent cells, and the cells were infected with* C. psittaci* serovar 6BC at an MOI of 1 in antibiotic-free complete media. The medium for infected cells was supplemented with 1 *μ*g/mL cycloheximide (Sigma, St. Louis, MO). Prior to the infection, the cells were pretreated with iron sulfate, INP0007 (ChemBridge, London, UK) or a MAPK inhibitor (Calbiochem, San Diego, CA).

### 2.3. Inclusion-Forming Unit (IFU) Detection

After the supernatants of the treated cells were collected, the adherent cells were fixed with methanol for 30 min at room temperature and permeabilized for 4 min at 4°C with 0.3% Triton X-100 in phosphate-buffered saline (PBS). After washing with PBS, the cells were incubated with a rabbit anti-*C. psittaci* 6BC antibody followed by incubation with a Cy2-labeled (green fluorescence) goat anti-rabbit immunoglobulin G (IgG) (Jackson ImmunoResearch Laboratories, USA) as the secondary antibody. All of the antibodies were diluted with 1% bovine serum albumin (BSA) in PBS, and the antibody incubations described above were preceded by intensive washes in PBS. After the final washing with water, the nuclei of the cells were stained using 4′,6-diamidino-2-phenylindole (DAPI, Sigma, USA). All of the images shown in this paper were captured under fluorescence microscopy (Zeiss Axioskop2). The inclusion bodies (green fluorescence) in the cells were counted under a microscope (at 40x magnification) to calculate the IFU/mL for each sample. Five fields were counted for each sample.

### 2.4. Cytokine Analysis

Q-PCR and ELISA were used to analyze the cytokine concentrations in the culture supernatants of the THP-1 cells. Total RNA was extracted from the THP-1 monolayer cells of each group using an SV Total RNA Isolation System (Promega, USA) according to the manufacturer's instructions. The extracted RNA was treated with DNase I (Promega, USA) for 30 min at 37°C and then for 10 min at 70°C to remove any DNA contamination. Two hundred nanograms of RNA were used as the template in the reverse transcription reaction. The total cDNA was obtained using reverse transcription PCR with the RevertAid First Strand cDNA Synthesis Kit (Fermentas, Canada) according to the manufacturer's instructions. Q-PCR was performed using the SYBR Taq kit (ABI, USA). The mRNA sequences for the target genes (IL-8, IL-6, TNF-*α*, IL-1*β*, and *β*-actin of* Homo sapiens*) were obtained from the GenBank database (http://www.ncbi.nlm.nih.gov/). *β*-Actin was used as an internal control. Specific primers were designed using Primer Premier 5.0 (Table 1) and synthesized by Shanghai Sangon Company. The Q-PCR reaction was performed on an Applied Biosystems model 7900HT Fast Real-Time PCR System in triplicate using 25 ng of cDNA and SYBR green Universal PCR Master Mix in a total volume of 25 *μ*L. The program consisted of an initial denaturation at 95°C for 10 min, followed by 40 cycles of 95°C for 15 s and 60°C for 1 min. The fold changes of the target genes were calculated using the 2^−ΔΔCT^ method [25].

The protein levels of IL-8, IL-6, TNF-*α*, and IL-1*β* in the supernatants of the treated cells were determined using ELISA kits (R&D Systems, USA) according to the manufacturer's protocols. The absorbance was measured at 450 nm with a Microplate Reader (Molecular Devices, USA), and the protein concentrations were calculated.

### 2.5. Signaling Pathway Assay

Western blot analysis was used to identify MAPK activation after* C. psittaci* infection. THP-1 cells infected with* C. psittaci* with and without pretreatment using MAPK inhibitors were harvested and homogenized with 2% sodium dodecyl sulfate (SDS) sample buffer. The samples were loaded onto 12% SDS polyacrylamide gels. After electrophoresis, the proteins were electrotransferred onto polyvinylidene difluoride (PVDF) membranes (Bio-Rad, USA). The membranes were blocked with 1% BSA in PBS overnight at 4°C, washed with PBS and incubated with rabbit monoclonal antibody against phospho-ERK, phospho-JNK, phospho-p38, ERK, JNK, and p38 (Cell Signaling Technology, USA) at 37°C for 2 h. These antibodies were diluted to 1 :  2000. After extensive washing with PBS, the membranes were incubated with horseradish peroxidase-conjugated goat anti-rabbit or anti-mouse IgG (Jackson ImmunoResearch Laboratories, USA) (diluted to 1 : 2000) at 37°C for 1 h. The detection was then performed using BeyoECL Plus (Beyotime, Shanghai, China). ERK, JNK, or p38 was used as the internal control, respectively. The supernatants of the THP-1 cells infected* C. psittaci* with or without pretreatment with MAPK inhibitors were collected. The relationship between the MAPK signaling pathways and the expression levels of inflammatory cytokines (IL-8, IL-6, TNF-*α*, and IL-1*β*) were analyzed by ELISA.

### 2.6. Statistical Analysis

Three independent experiments were performed. All of the data are presented as the mean ± standard deviation (SD). The statistical analysis was performed with one-way analysis of variance (ANOVA) followed by least squares difference (LSD) and Dunnett's T3 test using SPSS, version 12.0. A* P *value < 0.05 was considered statistically significant.

## 3. Results

### 3.1. Iron Sulfate Restored the* C. psittaci* Growth Defect Inhibited by INP0007

**Figure 1:**
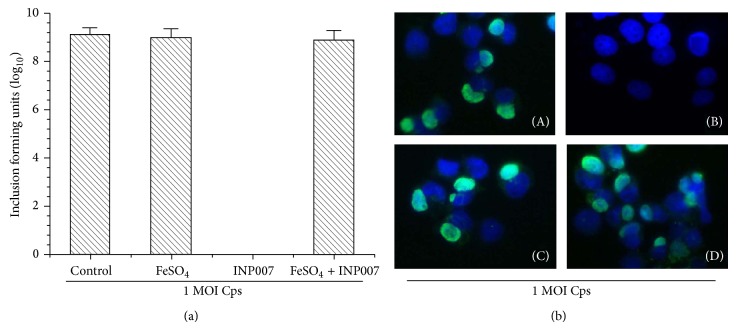
Inclusion-forming units (IFUs) of* C*.* psittaci* in THP-1 cells treated with iron sulfate, INP0007, or both. (a) Number of IFUs in each group. One million THP-1 cells were treated with 50 *μ*M FeSO_4_, 50 *μ*M INP0007, or both; the cells were then infected with* C*.* psittaci*. At 44 h p.i., the THP-1 cells were fixed and incubated with a rabbit anti-*C*.* psittaci* 6BC antibody and then incubated with a Cy2-labeled (green fluorescence) goat anti-rabbit IgG and DAPI. The inclusion bodies (green fluorescence) in the cells were counted under a fluorescence microscope (at 40x magnification). Five fields of each sample were counted. (b) Images of IFUs of* C*.* psittaci* in THP-1 cells. Cells infected with* C*.* psittaci* (A), cells infected with* C*.* psittaci* and pretreated with INP0007 (B), cells infected with* C*.* psittaci* and pretreated with FeSO_4_ (C), and cells infected with* C*.* psittaci* and pretreated with both INP0007 and FeSO_4_ (D) (40x magnification).

The differentiated THP-1 cells were treated with 50 *μ*M INP0007, 50 *μ*M FeSO_4_, or both and then infected with* C*.* psittaci* (MOI = 1). At 44 h after infection (p.i.), in the presence of the inhibitor 50 *μ*M INP0007, the THP-1 cells did not show evidence of* C*.* psittaci* infection (Figure 1(a)), and no inclusion bodies were detected (Figure 1(b)). The chlamydial growth deficit induced by INP0007 was completely reversed by the addition of 50 *μ*M FeSO_4_, and inclusion bodies were observed (Figure 1(b)).

### 3.2. The Expression of IL-8, IL-6, TNF-*α*, and IL-1*β* Induced by Chlamydial Infection Was Associated with a T3SS

**Figure 2:**
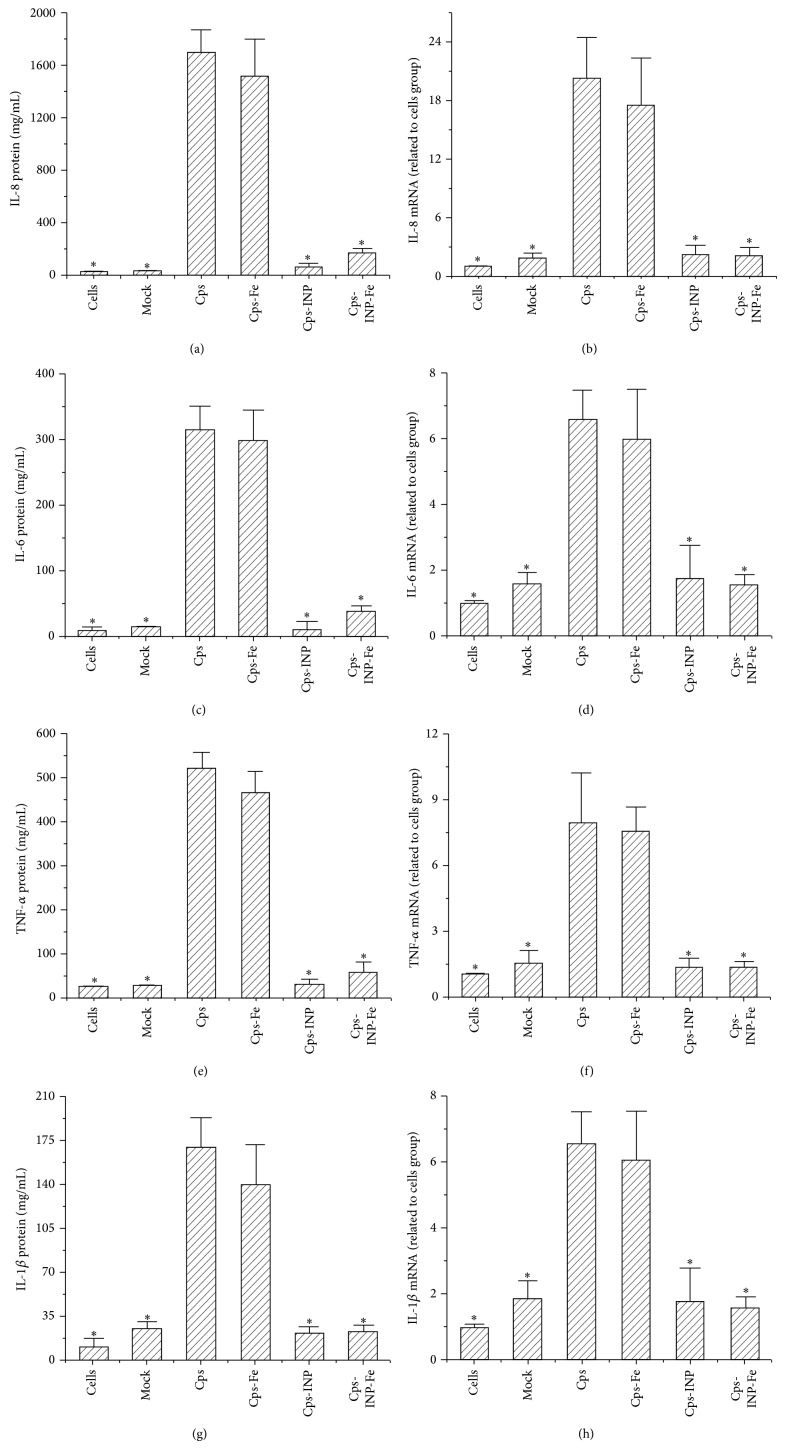
Production of IL-8, IL-6, TNF-*α*, and IL-1*β* in* C. psittaci*-infected THP-1 cells that were pretreated with iron sulfate (50 *μ*M FeSO_4_), 50 *μ*M INP0007, or both. THP-1 cells were infected with* C. psittaci* in the presence of 50 *μ*M INP0007 and/or 50 *μ*M FeSO_4_. Mock group: THP-1 cells were treated with heat inactivated Cps. At 44 h p.i., the levels of IL-8 (a), IL-6 (c), TNF-*α* (e), and IL-*β* (g) in the supernatants were assayed by ELISA. Total RNA was isolated from the THP-1 cells, and the transcription levels of IL-8 (b), IL-6 (d), TNF-*α* (f), or IL-*β* (h) were analyzed by Q-PCR. Cps, THP-1 cells infected with* C. psittaci*. Cps-Fe, THP-1 cells infected with* C. psittaci* and pretreated with FeSO_4_. Cps-INP, THP-1 cells infected with* C. psittaci* and pretreated with INP0007. Cps-Fe-INP, THP-1 cells infected with* C. psittaci* and pretreated with both FeSO_4_ and INP0007. ^*^ *P* < 0.05 compared with the Cps group.

At 44 h p.i., the protein levels of IL-8 (Figure 2(a)), IL-6 (Figure 2(c)), TNF-*α* (Figure 2(e)), and IL-1*β* (Figure 2(g)) in the supernatants of the differentiated THP-1 cells infected with* C. psittaci* in the presence of 50 *μ*M INP0007, 50 *μ*M FeSO_4_, or both were assayed using ELISA. The transcription levels of IL-8 (Figure 2(b)), IL-6 (Figure 2(d)), TNF-*α* (Figure 2(f)), and IL-1*β* (Figure 2(h)) in the treated THP-1 monolayer cells were analyzed using Q-PCR. At the transcriptional and protein levels, FeSO_4_ pretreatment did not statistically affect the expression of IL-8, IL-6, TNF-*α*, or IL-1*β* in the* C. psittaci*-infected THP-1 cells. Pretreatment with the T3SS inhibitor INP0007 significantly decreased the expression levels of these cytokines in* C. psittaci*-infected THP-1 cells with or without the addition of FeSO_4_. These data suggest that the increased expression of IL-8, IL-6, TNF-*α*, and IL-1*β* induced by chlamydial infection was associated with a T3SS.

### 3.3. The MAPK Signaling Pathway Is Activated by* C. psittaci* T3SS in THP-1 Cells

**Figure 3:**
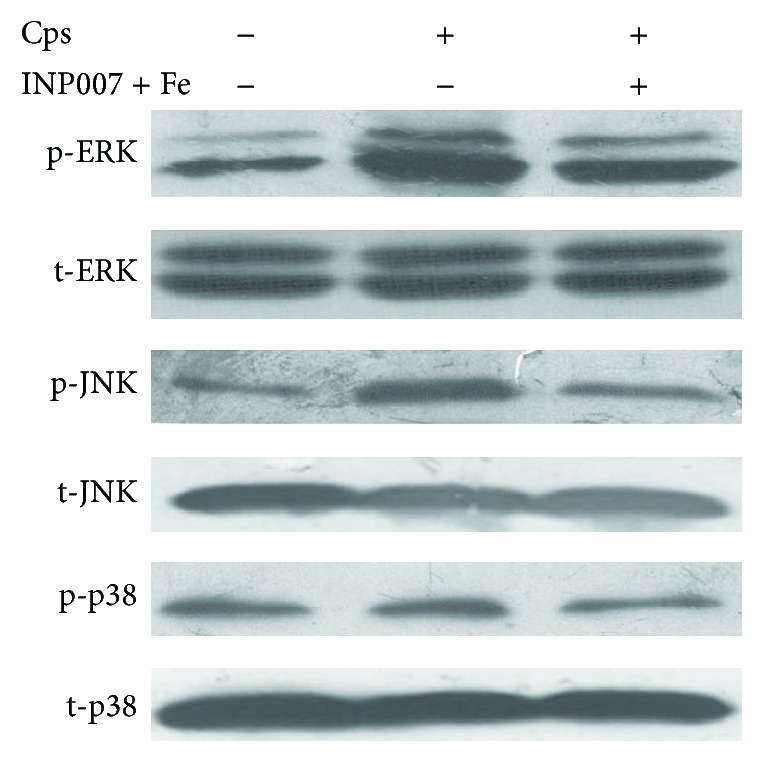
MAPK pathway assay of THP-1 cells infected with* C. psittaci* pretreated with iron sulfate and INP0007. The cells in each group were collected at 44 h p.i. The expression levels of p38, ERK, and JNK were analyzed by Western blot analysis. The expression levels of phospho-p38, phospho-ERK, and phospho-JNK were increased in the cells infected with* C. psittaci*. Iron sulfate and INP0007 pretreatment inhibited the increased expression. Cps, THP-1 cells infected with* C. psittaci*.

The phosphorylation levels of MAPKs (p38, JNK and ERK) were analyzed using Western blot analysis with specific antibodies at 44 h p.i. with* C. psittaci*. As shown in Figure 3,* C. psittaci *infection activated the ERK and JNK/MAPK signaling pathways, but not the p38/MAPK pathway. FeSO_4_ pretreatment did not affect the levels of phospho-ERK and phospho-JNK that were induced by* C. psittaci *infection. These data indicated that the T3SS increased the levels of phospho-ERK and phospho-JNK.

### 3.4. Inflammatory Cytokine Expression Induced by* C. psittaci* Infection via the ERK/JNK Signaling Pathway

**Figure 4:**
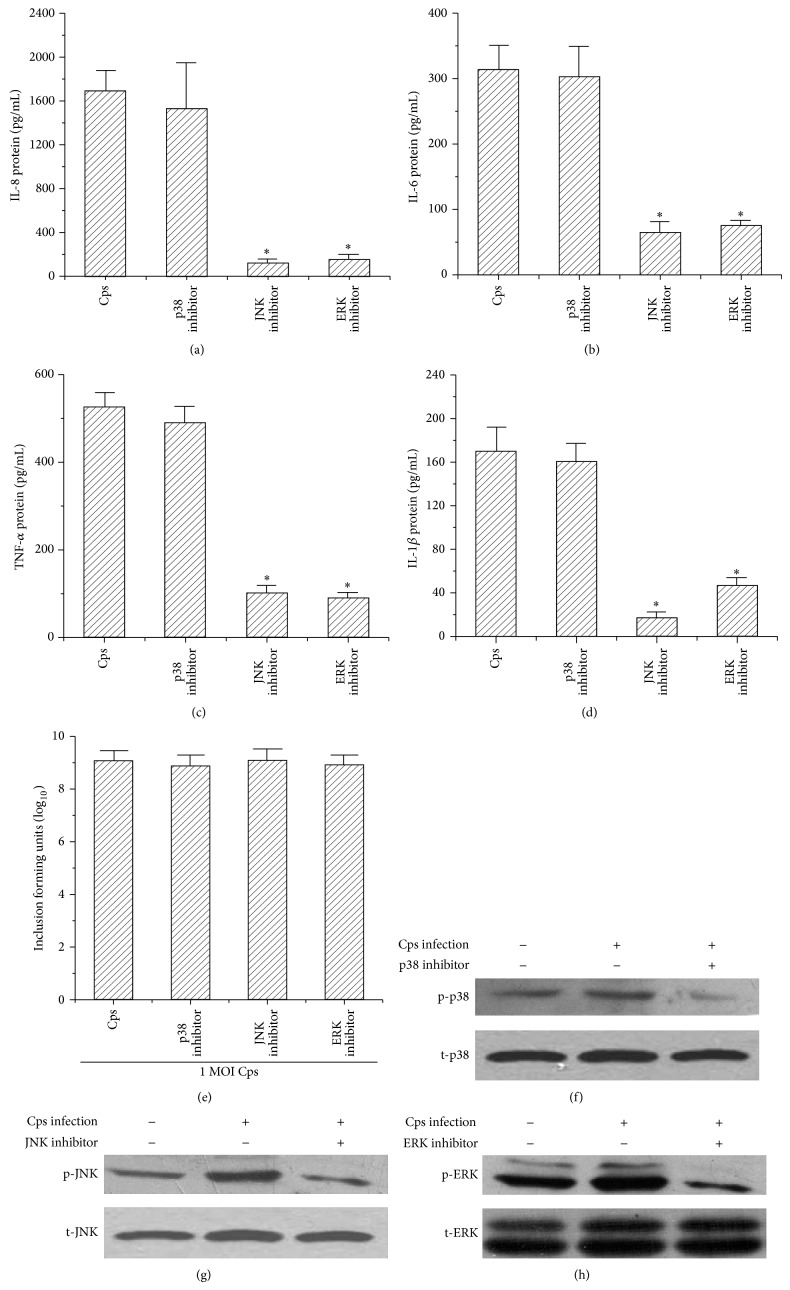
Production of inflammation cytokines and the MAPK pathway assay of* C. psittaci*-infected THP-1 cells treated with a p38, JNK, or ERK inhibitor. THP-1 cells were pretreated with a p38, JNK, or ERK inhibitor and infected with* C. psittaci*. The supernatants were collected, and the production of IL-8 (a), IL-6 (b), TNF-*α* (c), and IL-1*β* (d) was detected using an ELISA kit. Inclusion-forming units (IFUs) were counted by indirect immunofluorescence staining (e). The monolayer cells were harvested and homogenized. The levels of phospho-p38 (f), phospho-JNK (g), and phospho-ERK (h) were analyzed by Western blot analysis. These inhibitors did not inhibit the Cps growth. The JNK and ERK inhibitors decreased the production of IL-8, IL-6, TNF-*α*, and IL-1*β*; the p38 inhibitor did not decrease the production of these cytokines.  ^*^ *P* < 0.05 compared with the Cps group.

ERK or JNK/MAPKs inhibitors attenuated the increased levels of phospho-ERK, phospho-JNK, and phospho-p38 in* C. psittaci*-infected THP-1 cells (Figures 4(e), 4(f), and 4(g)). The protein levels of IL-8 (Figure 4(a)), IL-6 (Figure 4(b)), TNF-*α* (Figure 4(c)), and IL-1*β* (Figure 4(d)) were significantly decreased in the supernatants of* C. psittaci*-infected THP-1 cells by pretreated with a JNK or ERK inhibitor, but not by p38 inhibitor.

## 4. Discussion

In the present study, our study results demonstrated that* C. psittaci* infection enhanced the expression of IL-1*β*, IL-6, IL-8, and TNF-*α* and activated the ERK and JNK signaling pathways in THP-1 cells. The T3SS inhibitor INP0007 [19, 21], a small organic molecule that targets the T3SS of many human pathogens, prevented the growth of* C. psittaci* and decreased the expression of inflammatory cytokines. Exogenous iron restored the growth of* C. psittaci*; however, iron did not restore the expression inflammatory cytokines in infected THP-1 cells. ERK or JNK inhibition decreased the levels of inflammatory cytokinesin* C. psittaci*-infected THP-1 cells; however, p38 inhibition did not decrease the levels of these inflammatory cytokines. These results indicated that there was a correlation between the T3SS and the expression of these inflammatory cytokines. The ERK and JNK MAPK signaling pathways might play vital roles in the host inflammatory response induced by* C. psittaci* infection.

The T3SS, which is a complex bacterial structure, injects bacterial effector proteins into the host cell's cytoplasm and is required for the virulence and survival of several pathogens (e.g.,* Yersinia pestis*); the T3SS also plays an important role in the relationships between bacteria and the inflammatory responses of the host [26–28]. Small organic molecules targeting the T3SS apparatus of many human pathogens have been proposed as new antimicrobial agents; these molecules are called virulence blockers [29]. They can inhibit* Chlamydia* growth, development, and secretion, suggesting a role for the T3SS throughout the intracellular developmental cycle [30–35]. At all stages of infection, chlamydiae manipulate the host cells by secreting effector proteins that help establish a replicative vacuole and suppress innate immune response. Most of these effector proteins are likely T3SS effectors and are synthesized at all stages in the developmental cycle [36, 37]. Muschiol et al. reported that INP0400, an analogue of INP0007, could either block the entry of elementary bodies into host cells or inhibit the replication of reticulate bodies [31]. Previous studies have indicated that the T3SS inhibitor INP0007 can prevent the intracellular growth of* C. trachomatis* [31],* C. pneumoniae* [30], and* C. muridarum *[21] and decrease the host's inflammatory response [21] and inhibit the T3SS effector proteins expression and secretion [22, 33]. Interestingly, a study by Hudson et al. showed a decreased inflammatory response in a bovine intestinal ligated loop model of* S. enterica serovar Typhimurium *when the bacteria were preincubated with INP0007 [20]. Prantner and Nagarajan [21] reported that INP0007 could suppress the growth of* C. muridarum* and could suppress the production of inflammatory cytokines, including IL-1*β*, IL-6, and CXCL10. The addition of exogenous iron could restore the growth of* C. muridarum*; however, iron could not restore the expression of inflammatory cytokines in INP0007-treated mouse macrophages infected with* C. muridarum *[21]. Our study suggested that* C. psittaci *infection enhanced the expression levels of IL-1*β*, IL-6, IL-8, and TNF-*α* in THP-1 cells. INP0007 inhibited the growth of* C. psittaci* and decreased the expression of these inflammatory cytokines. Rescue of chlamydial growth by the addition of iron sulfate did not restore cytokine production. These results are consistent with the results of previous studies [28, 29]. Therefore, we inferred that the increased expression of several cytokines during infection was dependent on the T3SS, but not completely dependent on the growth of the bacteria.

MAPKs are important signaling pathways for the host immune response, and these pathways comprise a family of proline-directed serine/threonine kinases. Three major MAPKs, ERK, JNK, and p38, have been identified that transport extracellular signals to trigger intracellular responses [38, 39]. Previous studies have indicated that MAPKs are involved in the production of various proinflammatory cytokines during chlamydial infection [17, 20, 31]. When the ERK/MAPK pathway was blocked with 30 *µ*M PD98059, the levels of IL-1*β*, IL-6, IL-8, and TNF-*α* were reduced in THP-1 cells infected with* C. psittaci*. By blocking the JNK/MAPK pathway with 30 *µ*M SP600125, the expression levels of IL-1*β*, IL-6, IL-8, and TNF-*α* were decreased. The JNK/MAPK pathway appeared to be more sensitive to cytokine responses following* C. psittaci *infection. Blocking the p38/MAPK pathway with 30 *µ*M SB202190 did not change the proinflammatory cytokine levels. Our results also confirmed that these inhibitors did not affect the Cps growth. Therefore, we speculated that one or more T3SS effector proteins induced proinflammatory cytokine production in host cell by activating the MAPK signal pathways through phosphorylating variations of ERK and JNK.

The current study demonstrated that the chlamydial T3SS played an important role in the establishment of the host cytokine response, and the JNK or ERK MAPK signaling pathways were involved in the process. Further research should focus on understanding how INP0007 inhibits T3SS, how exogenous iron restores the growth of* C. psittaci* in host cells, and which chlamydial effector protein activates the JNK or ERK signaling pathways and initiates the host inflammatory response.

## Figures and Tables

**Table 1:** Primers used in the Q-PCR analysis.

Gene name	Forward primer	Reverse primer	Product (bp)	Accession number
*β*-Actin	GACTTAGTTGCGTTACACCCTTTC	CTGCTGTCACCTTCACCGTTC	161	NM_001101.3
IL-1*β*	CTACGAATCTCCGACCACCA	GGCAGGGAACCAGCATCTT	97	NM_000576
IL-6	AATAACCACCCCTGACCCAA	TTTGCCGAAGAGCCCTCA	147	NM_000600
IL-8	AGACATACTCCAAACCTTTCCACC	ACAACCCTCTGCACCCAGTT	156	NM_000584
TNF-*α*	AGCTGGAGAAGGGTGACCGA	CAGGGCAATGATCCCAAAGTA	99	NM_000594
